# Anatomic Landmarks for the Radial Tunnel

**Published:** 2008-06-22

**Authors:** Ron Hazani, Nitin J. Engineer, Arian Mowlavi, Michael Neumeister, W.P. Andrew Lee, Bradon J. Wilhelmi

**Affiliations:** Division of Plastic and Reconstructive Surgery, School of Medicine, University of Louisville, Louisville, KY

## Abstract

**Background:** The posterior interosseous nerve (PIN) can be difficult to locate within the radial tunnel. The deep branch of the radial nerve (DBRN) enters the supinator muscle after passing under the arcade of Fröhse. It courses through the superficial portion of the supinator muscle to exit distally as the PIN. Anatomic landmarks could facilitate diagnosis and treatment of radial tunnel syndrome and aid in the injection and decompression of the radial nerve. **Methods:** Eighteen cadaveric arms were used to identify anatomic landmarks to facilitate location of the PIN. The landmarks used include the palpable proximal radial edge of the radial head, proximally, and the mid-width of the wrist, distally. The skin was incised along this longitudinal line through the fascia. Deep within this plane the PIN was identified exiting the distal edge of the superficial portion of the supinator muscle. The proximal and distal edges of the supinator muscle were measured from the proximal radial aspect of the radial head. In addition, the course of the DBRN was appreciated proximal and distal to the superficial part of the supinator muscle. **Results:** The PIN was identified to exit the superficial part of the supinator muscle at an average distance of 7.4 ± 0.4 cm distal to the proximal radial aspect of the radial head. Distal to the distal edge of the supinator muscle, the PIN passed along a longitudinal vector from the radial head to the mid-width point of the wrist. From within the supinator muscle the DBRN courses retrograde in an oblique direction toward the lateral edge of the distal most part of the biceps tendon. **Conclusion:** The anatomic landmarks of the radial head and the mid-width of the dorsal wrist can be used to predict the course and location of the PIN. The DBRN can be predicted to enter the superficial part of the supinator muscle approximately 3.5 cm distal to the radial head, and the PIN is predicted to exit the supinator at 7.5 cm distal to the radial head.

Patients with radial tunnel syndrome and posterior interosseous nerve (PIN) syndrome can present with similar symptoms. Both have been implicated as a cause of resistant lateral elbow pain.^1^–^4^ The radial tunnel begins where the deep branch of the radial nerve (DBRN) courses over the radiohumeral (RH) joint. The tunnel ends where the DBRN becomes the PIN as it exits the distal edge of the superficial supinator.^5^ Structures within the radial tunnel that can cause compression of the DBRN include, proximally, fibrous fascial bands coursing superficial to the radial head; the radial recurrent artery and its vena comitans, also known as the leash of Henry; the fibrous edge of the extensor carpi radialis brevis (ECRB); the proximal edge of the supinator; and the distal edge of supinator muscle which is typically fibrous. The most common point of compression is the arcade of Fröhse,^6^ a fibrous arch at the proximal edge of the supinator.

Previous measurements of the DBRN use the supinator muscle and the radial nerve bifurcation as landmarks.^7^ Others use the radiohumeral joint line as a reference point for measurements of the distance between the radial nerve bifurcation to the proximal and distal edge of the supinator muscle.^6^ Molina and associates^8^ measured the length of the radial nerve within the supinator. In terms of a surgical approach, none have defined the entrance and exit points of the DBRN within the supinator in relation to superficial bony landmarks. The *purpose* of this study is to clarify these anatomic landmarks and guide clinicians in determining the location of injection sites for the diagnosis of radial tunnel syndrome or to facilitate a minimal incision approach for the treatment of this entity.

## METHODS

**Figure 1:**
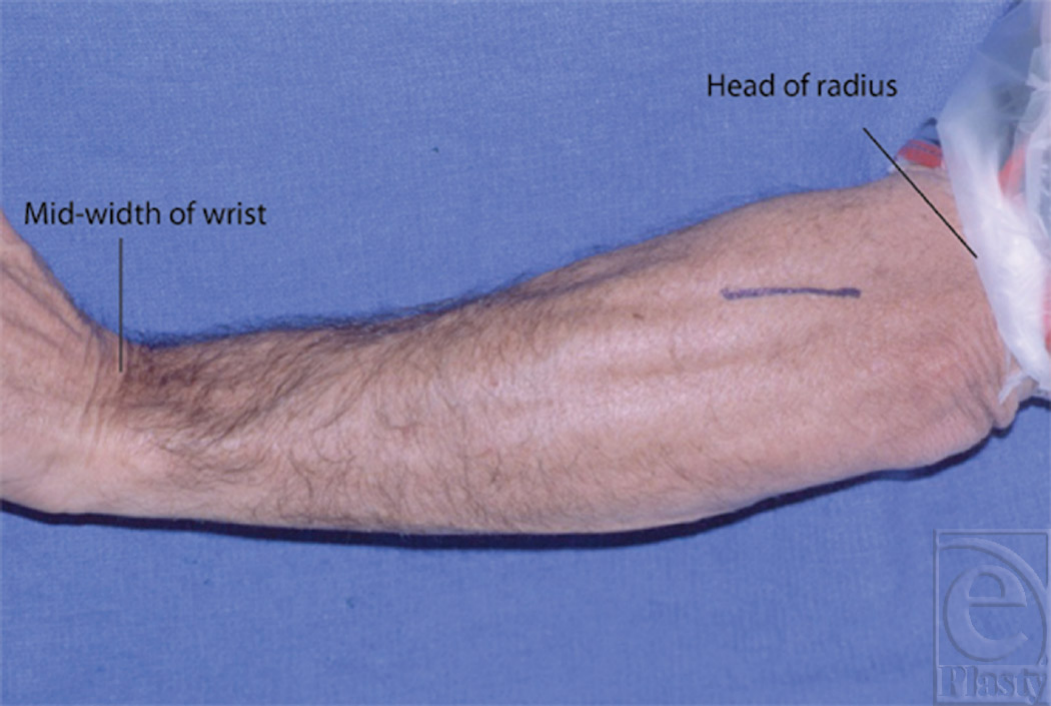
Skin incision on the basis of anatomic landmarks from the radial head of the radiohumeral joint along a line to the mid-width of the dorsal wrist.

**Figure 2:**
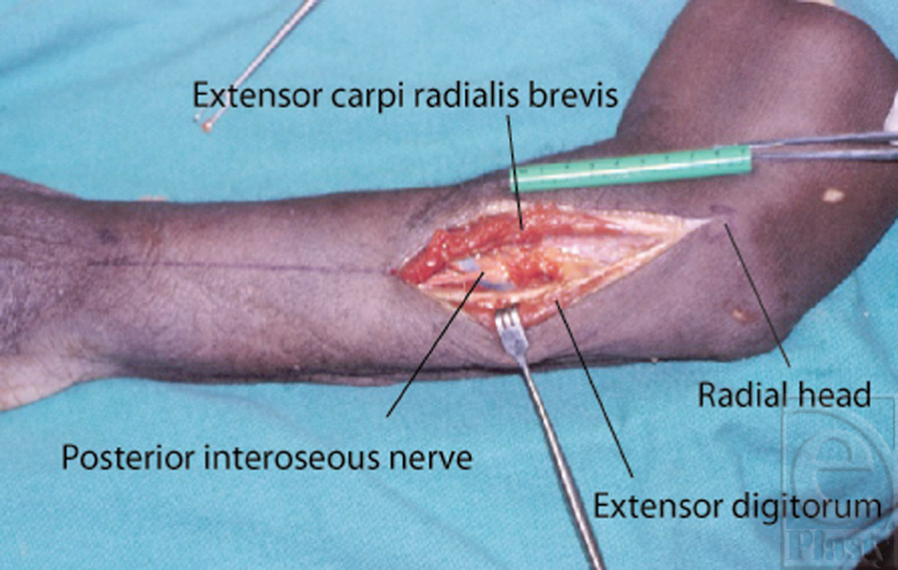
Measurements taken from the proximal edge of the radial head.

**Figure 3:**
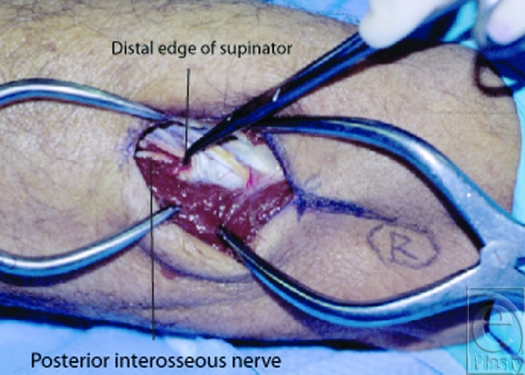
Fibrous edge of the distal supinator muscle.

The study design involved the dissection of 18 fresh cadaveric arms. The dissection was carried out via a posterior approach as the forearm was placed in a prone position. The skin was incised from the radial head of the RH joint along a line to the mid-width of the dorsal wrist (Fig 1). The DBRN was identified deep to the extensor compartment between the extensor digitorum communis (EDC) and ECRB muscles. The distance from the proximal radial head of the RH joint to where the DBRN enters and exits the supinator was measured for each limb (Fig 2). The supinator muscle's edges were examined for consistency and anatomic variations (Fig 3).

### RESULTS

The DBRN entered the supinator muscle, on average, 3.4 ± 0.3 cm distal to the head of the radius at the RH joint. The DBRN was found to exit the supinator muscle 7.4 ± 0.4 cm distal to the proximal head of the radius at the RH joint. After exiting the superficial supinator, the PIN courses along the vector from the head of the radius to the mid-width of the dorsal wrist. Within the supinator, the DBRN courses proximally in an oblique direction toward the lateral edge of the biceps tendon. e found the proximal supinator edge to be tendinous in 14 of the 18 dissections (78%), whereas the distal edge of the supinator was tendinous in 10 of the 18 arms (55%). No anatomic variants were noted throughout the study.

### DISCUSSION

*Radial tunnel syndrome* is a rare but debilitating cause of lateral elbow pain. The exact incidence of radial tunnel syndrome remains unclear; however, an epidemiological study of common compressive neuropathies demonstrates an incidence of 2.97 per 100,000 of new cases of radial neuropathy in men and 1.42 per 100,000 of new cases in women.^9^ When excluding trauma to the radial nerve, it appears that idiopathic entrapment of the nerve accounts for only 0.7% of the nontraumatic upper extremity lesions.^4^ The DBRN travels through the tunnel and innervates multiple extensors of the wrist and hand. Although compression of the radial nerve at that level is expected to produce motor symptoms, patients can sometimes develop significant pain localized to the extensor mass just below the elbow, separate from the lateral epicondyle. Grip weakness may also be present, probably secondary to pain.^5^

**Figure 4:**
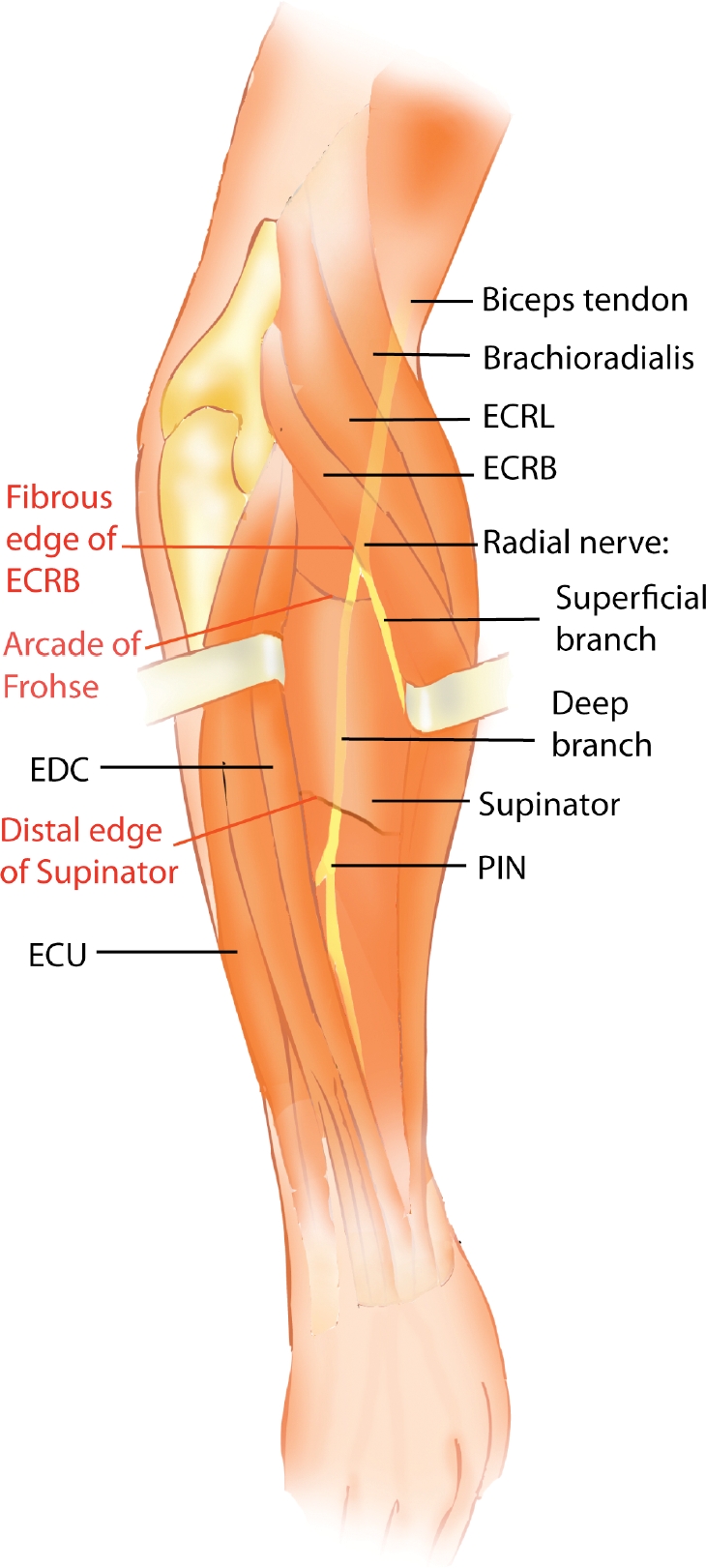
Illustration of the radial tunnel content through a dorsal approach demonstrating the bifurcation of the radial nerve into superficial and deep branches. The deep branch becomes the posterior interosseous nerve as it exits the distal edge of the supinator. Points of compression are marked in red. ECRB indicates extensor carpi radialis brevis; ECRL, extensor carpi radialis longus; ECU, extensor carpi ulnaris; EDC, extensor digitorum communis; and PIN, posterior interosseous nerve.

Inconsistency regarding the nomenclature of the motor branch of the radial nerve has led to overlap in the diagnosis of patients with radial tunnel syndrome and PIN syndrome. Anatomically, the radial nerve emerges from the supinator and then becomes the PIN. On the other hand, surgeons often refer to this area between the origin ofthe motor branch segment of the nerve and the distal edge of the supinator as the PIN. As clearly stated by Schnall and Wongworawat,^10^ we believe that standard anatomic definitions should be used to allow for clear communication among clinicians regarding the terminology of the radial nerve branches at the elbow (Fig 4).

There have been several approaches described to release the deep branch on the basis of the relationship of the incision to the brachioradialis muscle.^11^ Patients with radial tunnel entrapment may also present with tennis elbow.^2^,^12^ The posterior approach^13^,^14^ is recommended when both entities are present and require treatment. In addition to dual access to both the DBRN and the lateral epicondyle, other advantages of the posterior approach include easier identification of the PIN as it exits the supinator and a more acceptable, shorter scar as compared with one that is more visible anteriorly that might cross the antecubital fossa with the anterior approach. We found the dissection between the EDC and the ECRB, while staying on the EDC side of the septum, as the one that provides most exposure to the PIN, the superficial aspect of the supinator muscle, and the supinator muscle's edges.

Exposure of the radial tunnel region is inherently risky. Previous attempts to define the complex anatomy and the radial nerve distribution rely mainly on deep structures.^6^,^7^ The strength of this study is that it offers surgeons a consistent method of predicting the location of the deep branch in the radial tunnel before an incision is made. The proximal head of the radius at the RH joint is easily identified as it moves with pronation and supination of the forearm, thus allowing for clear measurements preoperatively.

Of the potential fibrous bands in the dorsal forearm, the superior border of the ECRB and the arcade of Fröhse have been implicated as the most likely structures to compress the DBRN.^15^ The tendinous consistency of the arcade ranges from 30% to 65% as measured by several investigators.^2^,^6^,^7^,^16^ Our cadaver dissections indicate an even higher measurement of up to 78%. In comparison, all of the 20 patients treated for radial tunnel syndrome in Lister's series^5^ had a thickened and fibrous arcade.

### CONCLUSION

Knowledge of the anatomic course of the DBRN can facilitate exposure and release of the compressed nerve. Anatomic landmarks have been described to identify the location of the radial nerve in the radial tunnel along a longitudinal vector from the radial head to the midline of the wrist. From the distal lateral edge of the biceps tendon, the DBRN courses obliquely to enter the proximal supinator edge 3.4 cm from the RH joint. The DBRN courses through the supinator to exit as the PIN at approximately 7.5 cm distal to the RH joint. Mapping of the expected location and path through which the DBRN traverses across the supinator muscle can serve clinicians in planning a reliable surgical approach to radial tunnel release.
